# Squarate Cross-Linked Gelatin Hydrogels as Three-Dimensional Scaffolds for Biomedical Applications

**DOI:** 10.1021/acs.langmuir.1c02080

**Published:** 2021-11-22

**Authors:** Simone Stucchi, Danilo Colombo, Roberto Guizzardi, Alessia D’Aloia, Maddalena Collini, Margaux Bouzin, Barbara Costa, Michela Ceriani, Antonino Natalello, Piersandro Pallavicini, Laura Cipolla

**Affiliations:** † Dept. of Biotechnology and Biosciences, University of Milano-Bicocca, P.zza della Scienza 2, 20126 Milano, Italy; ‡ Dept. of Physics “Giuseppe Occhialini”, University of Milano-Bicocca, P.zza della Scienza 3, 20126 Milano, Italy; § Nanomedicine Center, University of Milano-Bicocca, P.zza della Scienza 3, 20126 Milano, Italy; ∥ Dept. of Chemistry, Università degli Studi di Pavia, Viale Taramelli 12, 27100 Pavia, Italy

## Abstract

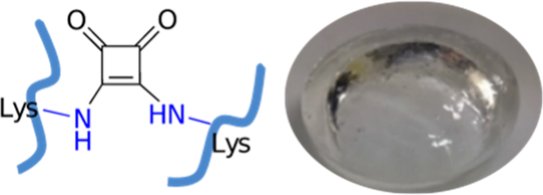

Hydrogels are useful platforms as three-dimensional (3D) scaffolds for cell culture, drug-release systems, and regenerative medicine applications. Here, we propose a novel chemical cross-linking approach by the use of 3,4-diethoxy-3-cyclobutene-1,2-dione or diethyl squarate for the preparation of 5 and 10% w/v gelatin-based hydrogels. Hydrogels showed good swelling properties, and the 5% gelatin-based hydrogel proved suitable as a 3D cell culture scaffold for the chondrocyte cell line C28/I2. In addition, diffusion properties of different sized molecules inside the hydrogel were determined.

## Introduction

In recent years, hydrogels have become popular as three-dimensional (3D) scaffolds for cell culture providing robust platforms for investigating cell physiology,^1−3^ pathology,^4^ tissue regeneration,^5,6^ drug discovery,^7^ and delivery.^8^

Depending on the chemistry of the polymeric constituents, and of the cross-linking strategy, hydrogels show different physico-chemical and biological features, accompanied by peculiar advantages and limitations. In this framework, the search for new hydrogels and cross-linking strategies is still ongoing, in order to ameliorate their performances toward the desired application. The increasing need for advancements in hydrogels as robust platforms as 3D cell culture scaffolds (i.e., for cell therapies, tumor models, drug delivery systems, and tissue engineering) is prompting the research in the field, that is expected to boost the market growth in the next years (compound annual growth rate (CAGR) of 10.7% from 2021 to 2028, https://www.grandviewresearch.com/industry-analysis/3d-cell-culture-market).

Among natural polymers, gelatin, obtained from collagen hydrolysis, is an attractive candidate for hydrogel preparation since it is biocompatible, possesses cell-adhesive properties, has limited costs, and it is easily accessible.^9,10^ However, gelatin is featured by poor mechanical properties, being water soluble at 37 °C. The mechanical properties of gelatin can be improved with different cross-linking agents, that is, exploiting the chemistry of the amino acid side chains through suitable cross-linkers (i.e., glutaraldehyde, genipin, and dextran dialdehyde) and chemistries (thiol–ene, Michael addition, Huisgen cycloaddition, carbodiimide chemistry, epoxy chemistry, etc.).^11−13^

Since the behavior of the hydrogels is mainly due to the functional groups present in the polymer, new cross-linking agents and chemistries are desired. In this work, the properties of gelatin-based hydrogels obtained by the use of 3,4-diethoxy-3-cyclobutene-1,2-dione or diethyl squarate (DES) as a homobifunctional cross-linker were investigated. To the best of our knowledge, DES has not been used before as a cross-linking agent for gelatin hydrogel preparation.

## Results and Discussion

### Hydrogel Synthesis and Physico-Chemical Features

**Figure 1:**
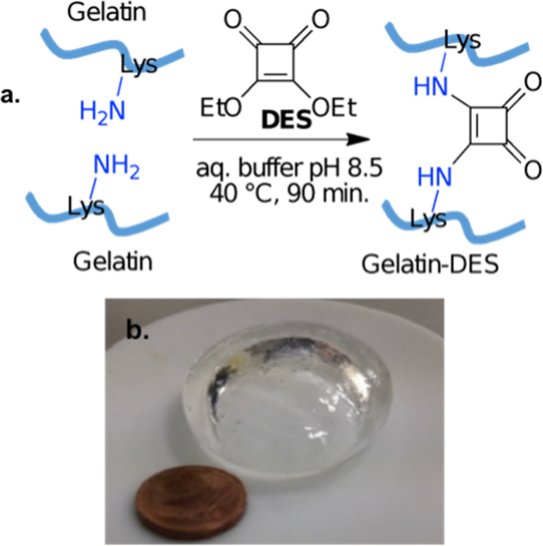
(a) Homo cross-linking of gelatin by 3,4-diethoxy-3-cyclobutene-1,2-dione (DES) and (b) gelatin cross-linked hydrogel.

Squaric acid diesters, such as DES, are useful reagents for amino-functional compound coupling and bioconjugation reactions.^14^ Porcine skin gelatin contains 27 lysine residues per 1000 amino acids^15^ and can be efficiently exploited for homo cross-linking reactions by DES. Thus, 5% w/v and 10% w/v gelatin in buffer solution at pH 9.3 (5% Gel-DES and 10% Gel-DES, respectively) were reacted with DES as the cross-linker at 40 °C for 90 min (Figure 1).

**Figure 2:**
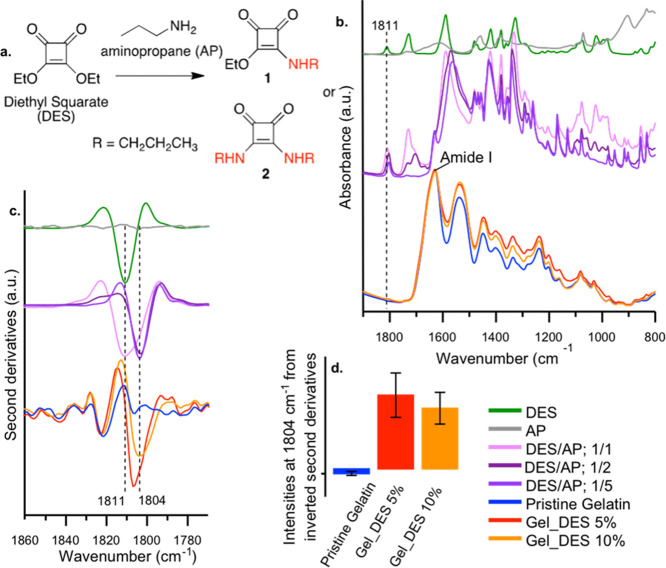
(a) Reaction between DES and AP; (b) FTIR spectra in the 1900–800 cm^–1^ region; (c) second derivatives of spectra collected in “b”; and (d) relative intensity of the 1804 cm^–1^ band.

The cross-linking to the bisamide product was checked by Fourier transform infrared (FTIR) spectroscopy (Figure 2). In order to validate the ability of this spectroscopic approach to discriminate between squarate esters and squaramides, control experiments using 1-aminopropane as model amine were conceived (AP, Figure 2a), using 1:1, 1:2, and 1:5 DES/AP molar ratios. It is well known that the treatment of squaric acid diesters with a primary or a secondary amine at room temperature in a 1:1 M ratio produces the corresponding squaric acid esteramide (i.e., monoamide **1**, Figure 2a) without the formation of the bisamide^14^ (i.e., compound **2**, Figure 2a); on the contrary, the bisamide can be obtained reacting DES with at a 1:2 DES/amine ratio at least. The selective formation of the monoamide is explained by the much faster amidation of the diester compared to the resulting ester amide. This allows the selective and sequential amidation if additional amine is added to the ester monoamide. DES FTIR absorption and second derivative spectra display a characteristic band around 1811 cm^–1^, corresponding to the C=O stretching vibration of DES carbonyl esters,^16^ whereas AP has no peaks in this spectral region (Figure 2b–d). The conversion of the ester carbonyls to squaramides induces a shift of this peak down to around 1804 cm^–1^ as can be better appreciated in the second derivative spectra, whose minima correspond to absorption maxima (Figure 2b,c). Furthermore, the second derivative analysis of the FTIR absorption spectra allows us to discriminate between squarate esters and squaramides and in particular between monoamide and bisamide. Indeed, the conversion of the ester carbonyls to squaramides induces a shift of the IR peak from 1811 cm^–1^ to around 1804 cm^–1^. Based on these observations, FTIR spectra of pristine and cross-linked gelatin were recorded. The absorption spectra of pristine gelatin, 5% Gel-DES, and 10% Gel-DES are dominated by the Amide I band due to the C=O stretching vibration of the protein peptide bond (Figure 2b), which is sensitive to the protein secondary structures;^17^ moreover, the three gelatin samples display superimposable Amide I band indicating comparable overall secondary structures (Figure 2b). The absorption spectra of 5% Gel-DES and 10% Gel-DES show a weak band around 1804 cm^–1^, absent in pristine gelatin. This component can be better appreciated in the second derivative spectra (Figure 2c,d). The presence of this peak in the spectral region of the bisamide absorption confirms the effectiveness of gelatin cross-linking by DES.

**Figure 3:**
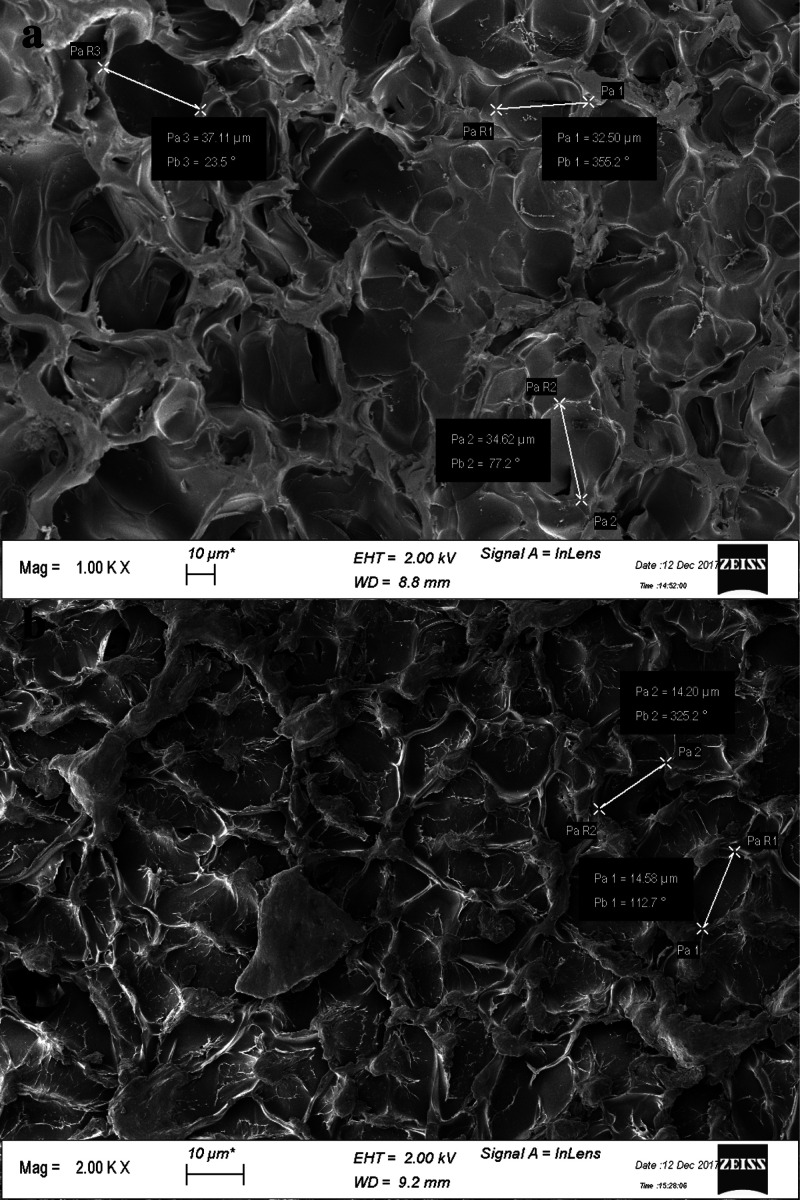
SEM images of the superficial portion of (a) Gel-DES 5% and (b) Gel-DES 10%.

SEM analysis of freeze-dried gelatin hydrogels showed 5% Gel-DES hydrogel featuring larger pores, if compared to the 10% Gel-DES samples, as it may be expected: 5% Gel-DES possesses an average diameter of the pores of 31.8 ± 4.8 μm, roughly three times higher than 10% Gel-DES, featured with 12.0 ± 2.4 μm pore sizes (Figures 3 and S1 in Supporting Information, respectively).

**Figure 4:**
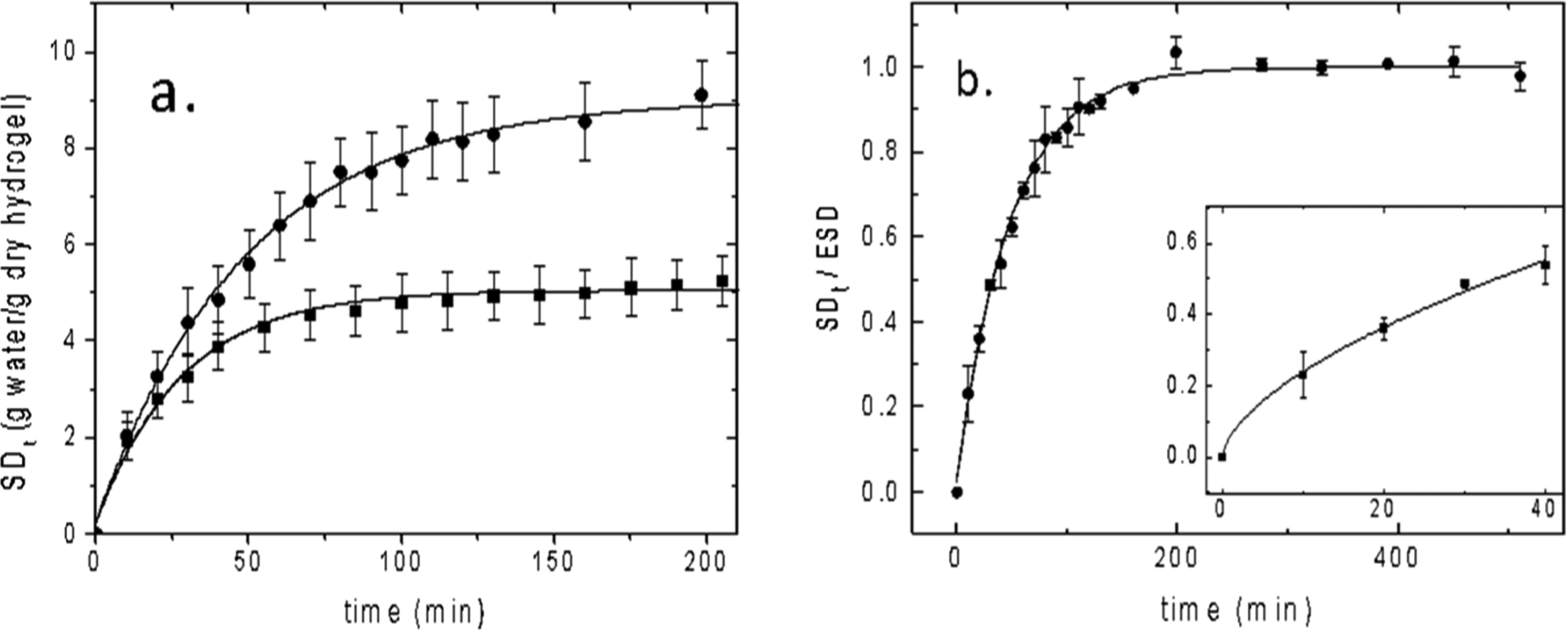
(a) 5% Gel-DES (full circles) and 10% Gel-DES (full squares) SD_*t*_ vs time, full lines are best fit to eq 2; (b) 5% Gel-DES swelling curves normalized to ESD as a function of time, the continuous line is the fit to eq 2. Inset: zoom on the early stages of the swelling kinetic. The continuous line is the best fit according to eq 4.

Freeze-dried gelatin specimens were also characterized for their swelling abilities. Gel swelling properties are usually dependent upon several factors, including pore size of the network, interactions within the network (polymer chains and cross-linkers), solvent, and chain mobility during the swelling process.^18^ Cross-linked gelatin (1 g) was placed into 40 mL of deionized water, and weight increase was measured over time till equilibrium was reached.^19^ Data are presented in terms of swelling degree (SD_*t*_) according to eq 1 (see the materials and methods section) in Figure 4a. 5% Gel-DES shows an equilibrium swelling degree (ESD) = 9.9 ± 1.0 (g of water absorbed)/(g of dry hydrogel), while the 10% Gel-DES reaches an ESD = 6.3 ± 0.7 (g of water absorbed)/(g of dry hydrogel), as determined by the best fit of the data versus time using eq 2 shown in the panel as a full line. Coherently, the percentage water content at the equilibrium (EWC) defined in eq 3 is 90.0 ± 1% for the 5% Gel-DES and 84.1 ± 1.7 for the 10% Gel-DES.

For the 5% Gel-DES sample, the swelling kinetic has been analyzed versus time in more detail: Figure 4b shows the normalized kinetic curve SD_*t*_/ESD versus time in minutes. The characteristic time is 50 ± 3 min (the best fit using eq 2 is shown in the panel as a full line), while by fitting the early swelling time only (SD_*t*_/ESD < 0.6) to eq 4, an exponent of *n* = 0.64 ± 0.05 has been obtained corresponding to anomalous (non-Fickian) solvent diffusion (Figure 4b, inset).

In summary, the two hydrogels differ in their water-retaining abilities: the higher the pore size (lower gelatin concentration and lower ratio of gelatin:cross-linker), the higher the EWC (and the ESD).

### Hydrogel Biological Assays

**Figure 5:**
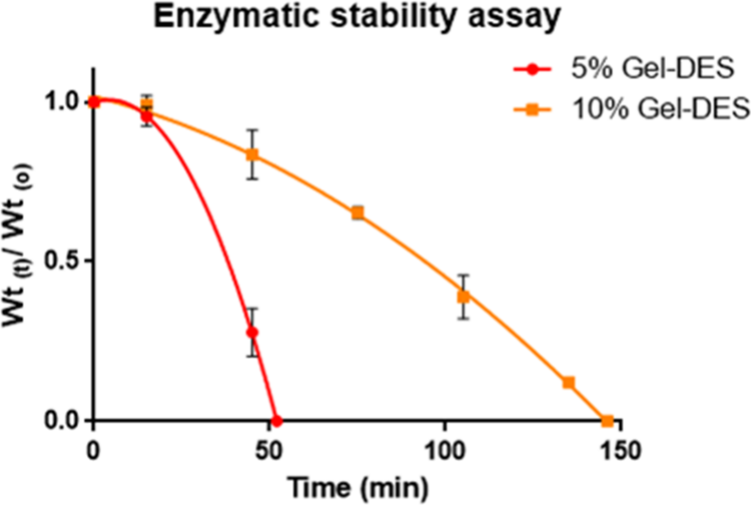
5% Gel-DES and 10% Gel-DES enzymatic degradation by collagenase type I from *C. histolyticum*.

Aiming at assaying the synthesized hydrogels as 3D cell culture scaffolds, resistance to enzymatic degradation was tested by collagenase. Freeze-dried 5% Gel-DES and 10% Gel-DES specimens (1 g) were treated with *Clostridium histolyticum* type I collagenase, and degradation was determined as weight loss as a function of time (Figure 5). 5% Gel-DES was fully digested by collagenase in 50 min, while 10% Gel-DES required almost 150 min for complete degradation.

In order to evaluate the biocompatibility of the gelatin-based hydrogels, immortalized C28/I2 chondrocytes were chosen as relevant cell lines, due to the great interest in hydrogels for cartilage regeneration,^20−22^ and compared with HEK293 cells, featured by their robustness, ease of growth, and reliability in cell experiments, frequently applied as a model for toxicity and biocompatibility tests of new biomaterials.^23−25^ Interestingly, the behavior of the two cell lines gave different results, both in terms of adhesion and cell morphology.

**Figure 6:**
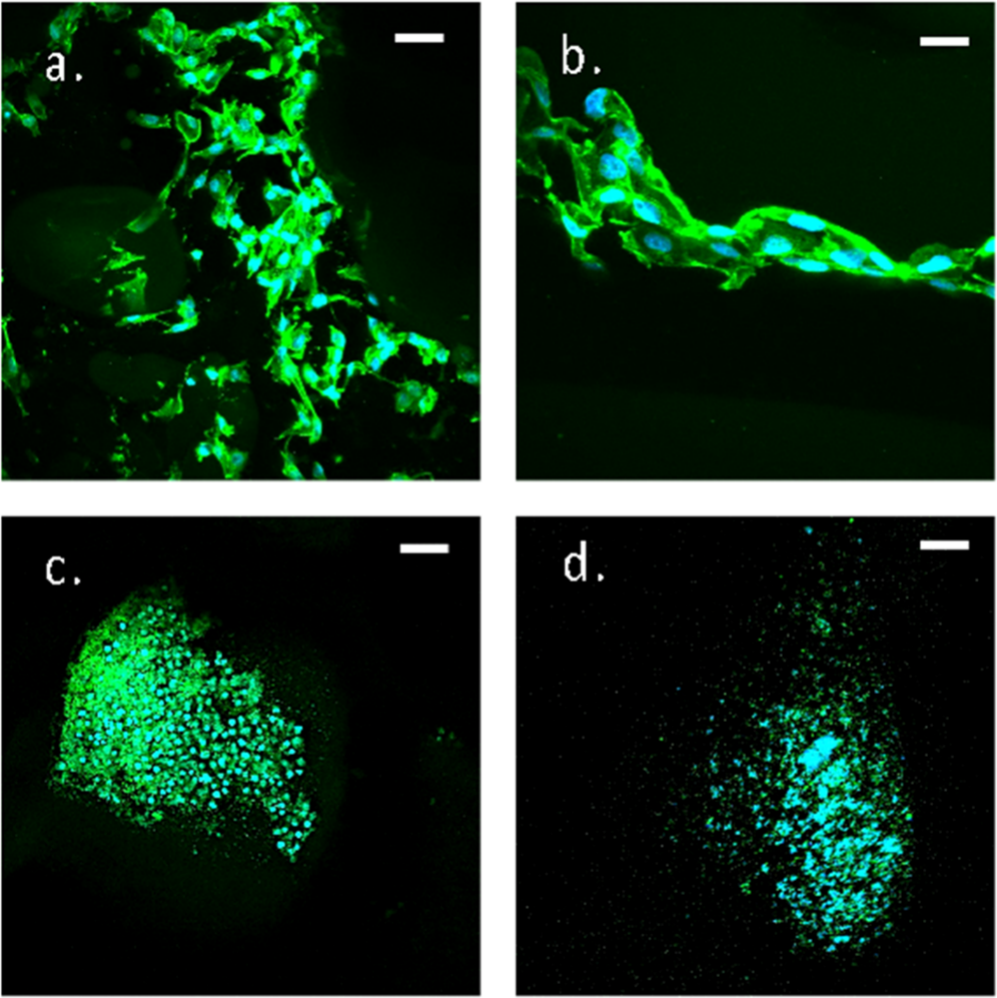
Cell colonization of 5% Gel-DES: DAPI emission at 485/30 nm is shown in cyan, and TRIC emission at 600/40 nm is shown in green. Panels a, b: C28/I2 chondrocytes; panels c, d: HEK293 cells. Bar size is 57 μm for panels (a,c), 28 μm for panel (b), and 23 μm for panel (d).

Freeze-dried hydrogels were placed in a 24-well plate, and C28/I2 or HEK293 cell suspensions (5 × 10^6^ cells/mL) were incubated for 4 h at 37 °C with 5% CO_2_ and cultured for 2 weeks. In order to evaluate the cell morphology, adhesion, and spreading, hydrogels were cut into sections and stained with TRITC-Phalloidin and DAPI for cytoskeleton and DNA, respectively, and imaged on a two-photon excitation confocal microscope, as shown in Figure 6. C28/I2 cells showed good adhesion properties and elongated morphology when grown in the 5% Gel-DES (Figure 6a,b) in contrast to the 10% Gel-DES (Supporting Information, Figure S2). HEK293 cells poorly adhered to the pore surface (Figure 6c,d): cells generated cell clusters, as normally epithelial cells do, but they acquired a rounded morphology, suggesting that cells do not appreciate the support. In fact, HEK293 cells seem to prefer cell–cell instead of cell–hydrogel adhesion. Thus, the 5% Gel-DES resulted in the best suited cell culture hydrogel for the C28/I2 cell line, while none of the two hydrogels promoted HEK293 cell adhesion. These observations confirm the need to fine tune hydrogels for both the specific cell line and the desired application. Given the better performance of 5% Gel-DES with chondrocytes, only this hydrogel was further characterized in terms of diffusion properties of molecules of different sizes.

### Hydrogel Diffusion and Delivery Properties

#### Uptake and Release Studies

Uptake and release of a small fluorescent molecule were investigated in the 5% Gel-DES. In order to study diffusivity and release properties, rhodamine 6G (F.W. 479 Da) was chosen as a model compound, due to its absorption and fluorescence properties allowing easy detection.

Rhodamine uptake kinetic was studied spectrophotometrically by soaking 5% Gel-DES in Rhodamine-6G solution, as described in detail in the experimental section.

**Figure 7:**
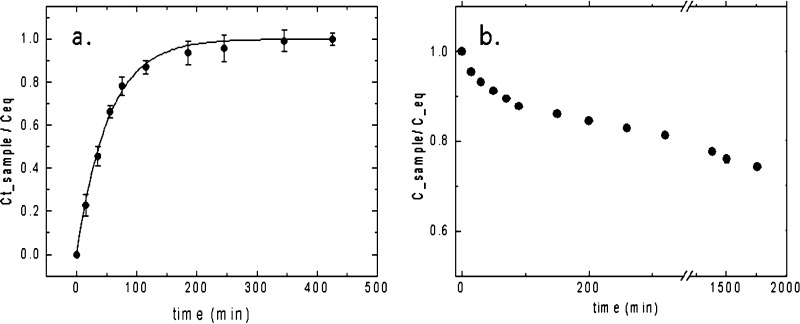
(a) Rhodamine uptake expressed as the ratio between the concentration at time *t* and the equilibrium concentration as a function of time (black line is the fit curve); (b) Rhodamine release normalized to equilibrium concentration as a function of time.

The hydrogel reached an equilibrium uptake of 64 ± 2% of the initial dye concentration (7.0 ± 0.15 μM); the loading kinetics is reported in Figure 7a as a plot of the loaded dye normalized to the equilibrium value C_eq_ = 4.5 ± 0.1 μM. The kinetic behavior can be analyzed according to the Berens-Hopfenberg model.^26,27^ The characteristic uptake time is τ = 55.6 ± 4 min, close to the characteristic swelling time of the hydrogel (Figure 4b).

The dye release kinetics was also investigated by soaking the rhodamine-loaded hydrogel in 3 mL of water, refreshed every 15 min. From the solution absorbance, the concentration of released rhodamine at each time point was estimated (Figure 7b). The kinetics showed that dye release from the hydrogel is very slow, reaching equilibrium at long times (>1000 min), corresponding to 25 ± 2% released dye. In summary, while rhodamine loading resulted fairly good (over 60% of compound is loaded into the hydrogel), its release is limited (only 25% of compound release over 1000 min). Given the slow release kinetics, the sample compound is not diffusing out of the gel, unless metabolic degradation of gelatin occurs, offering some advantages in view of the releasing process for medical applications.

#### Diffusion Studies

Diffusion studies were performed with three different probes, differing in size and/or molecular weight, such as rhodamine 6G (479 Da), green fluorescent protein (GFP, 27 kDa, 4 nm × 2 nm in size), and 20 nm diameter fluorescent polystyrene nanobeads.

Probe diffusion within the 5% Gel-DES hydrogel was characterized by the fluorescence correlation spectroscopy (FCS) technique. By detecting the fluorescence fluctuation in the observation volume (few cubic microns) of a low concentration fluorescent probe (typically few tens of nanomolar concentration), it is possible to estimate diffusion rates due to Brownian motion by the fluorescence autocorrelation function (ACF), as detailed in the material and methods section.

Typically, 200 μL of 5% hydrogel was directly cast in a multi-chamber cover slide and incubated in the probe solution at the proper concentration. After 4 h of incubation, the FCS ACFs have been acquired and compared with the curves obtained for the same probe in solution.

The diffusion behavior of the probes is resumed in Table 1.

**Table 1:** Diffusion Coefficient for Probes Used for the Study in the 5% Gel-DES Hydrogel

probe	F.W. (kDa)	dimension (nm × nm)	diffusion in solution (μm^2^/s)	diffusion in hydrogel (μm^2^/s)	*D*_sol_/*D*_hydr_
rhodamine 6G	0.479		300	40 ± 4	7.5±0.9
GFP	27	4 × 2	90 ± 2	13 ± 2	6.9±1.1
polystyrene nanobeads	n.a.	20 (diameter)	0.4	0	n.a.

The 300 mμ^2^/s rhodamine diffusion coefficient in solution has been used to determine the beam waist parameter *w*_0_ that has been kept fixed in the ACF fitting in the other cases.

**Figure 8:**
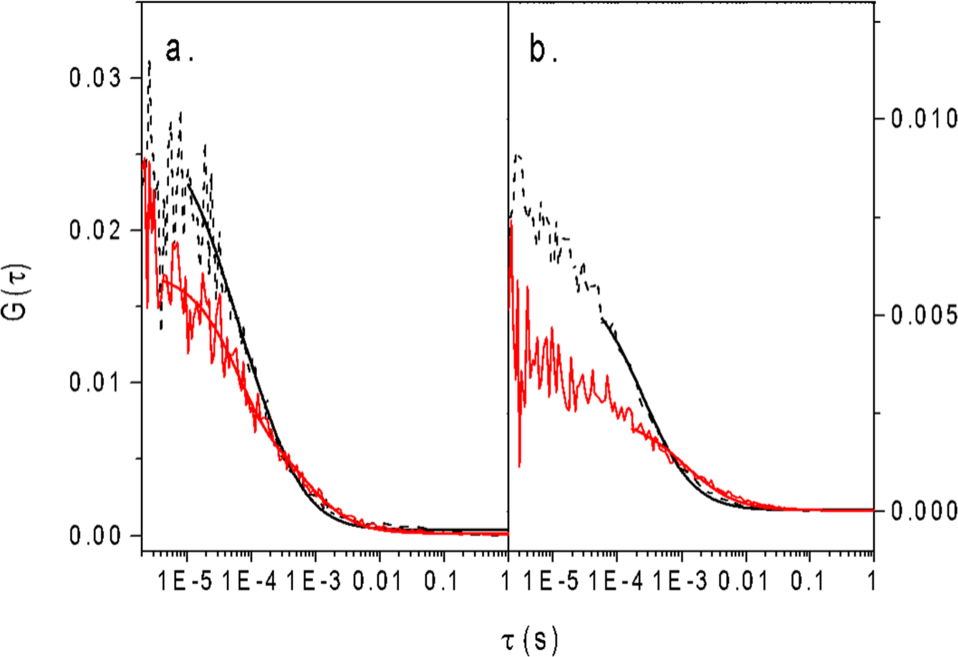
Rhodamine diffusion in 5% Gel-DES. (a) ACF average curve vs time lag for Rhodamine 6G in water (dotted, black) and in 5% Gel-DES (solid, red); (b) ACF average curve vs time lag for GFP in phosphate buffer (dotted, black) and in 5% Gel-DES (solid, red). The continuous lines represent the best fit with eq 5 in both panels.

The ACFs in hydrogel (continuous lines) and in aqueous solution (dashed lines) are reported in Figure 8. The ACFs obtained in the hydrogel samples are shifted to longer lag times with respect to the curves obtained in water (or buffer) solutions, suggesting a longer diffusion time for the fluorescent probes. By fitting the ACFs diffusive part with eq 6 (Figure 8, FCS, continuous lines), the diffusion coefficient of rhodamine in hydrogel was found to be *D*_hydr_ = 40 ± 4 μm^2^/s to be compared with the value of 300 μm^2^/s in water. GFP diffusion coefficient in the hydrogel is *D*_hydr_ = 13 ± 2 μm^2^/s to be compared with the value of *D*_sol_ = 90 μm^2^/s in solution, as reported in the literature.^28^

Data suggest that hydrogel affects the diffusion of both Rhodamine 6G and GFP at the same extent within the experimental uncertainties, showing that Brownian diffusion is not hindered by the gel pores: in both cases, the diffusion rate is almost sevenfold slower than that in solution (the ratio of the diffusion coefficients in the hydrogel and in solution for the two probes is 7.2 ± 0.6). This observation suggests that the same diffusion mechanism is involved for both probes, and that the hydrogel decreases the diffusion of the probes by the same amount, but the Brownian diffusive model still holds.

Release and diffusion data indicate that the squarate cross-linked hydrogel efficiently allows small molecule diffusion among its pores, and it does not leak the loaded compound in solution, thus being an interesting platform for further studies of drug delivery after *in vivo* controlled degradation.

A different behavior is found when 20 nm fluorescent nanobeads were incubated with the hydrogel. After 4 h of incubation, a fluorescence signal could be detected on the chamber bottom, indicating that the nanobeads sedimented through the hydrogel. However, FCS (as used for the previous measurements) did not afford reliable ACFs since no appreciable fluctuations of the fluorescence signal could be detected on a microsecond—hundreds of millisecond time scale. Therefore, fluorescence fluctuation kinetics has been probed on longer times, by acquiring image stacks using a confocal scanning microscope. Small fields of view (∼10 μm) have been scanned repeatedly up to several minutes. The superposition of the images shows that the beads can be considered immobile in the time scale considered. Since their diffusion coefficient in aqueous solution is about *D*_sol_ = 0.4 μm^2^/s, a seven time decrease in the diffusion coefficient would give characteristic times shorter than 200 ms, detectable with a minute duration measurement. In summary, larger probes (as fluorescent nanobeads) are able to slowly penetrate into the gel matrix; however, the sedimentation motion prevails on the Brownian motions, preventing further movements once the bottom of the chamber is reached. Therefore, the diffusion in the 5% Gel-DES hydrogel appears to be size-dependent, allowing sedimentation but hindering free diffusion even if the average pore size is much larger than the bead size.

Different nanoparticles, that is, gold nanostars (GNSs), have also been investigated as potential smart materials to be incorporated within the hydrogel. These plasmonic anisotropic nanoparticles (about 50 nm × 10 nm of branch size) are able to absorb light in the near-infrared (NIR) region and convert the absorbed energy into heat, through a pronounced photothermal effect.^29^ The photothermal effect exerted by similar nanoparticles is finding applications, for example, in tumor treatment and controlled drug delivery.^30^ In addition, when a pulsed laser beam in the NIR is focused on a suspension containing GNS, two photon excitation can occur, and a strong luminescence is emitted from the nanoparticles in the visible region of the electromagnetic spectrum to an inter-band conversion, thus allowing their detection.

Hence, GNSs have been incorporated into the hydrogel during its preparation, and their behavior has been investigated. In fact, it was previously confirmed that GNSs do not diffuse inside the hydrogel after hours of incubation as occurred for the 20 nm beads; their diffusion is hindered probably by their branching structure and larger size, as proved by confocal scanning microscope imaging of the loaded hydrogel after 24 of swelling in water in the reflection mode. After repeated time stacks, no motion of the nanostars is detected (data not shown).

However, when the photothermal effect is exploited, GNSs are able to diffuse within the gel. The diffusion has been visualized by coupling to the same setup used for FCS measurements using an electron multiplying charge-coupled device (EMCCD) camera in order to obtain stacks of images acquired for almost 10 s with typical frame rates about 30 fps.

**Figure 9:**
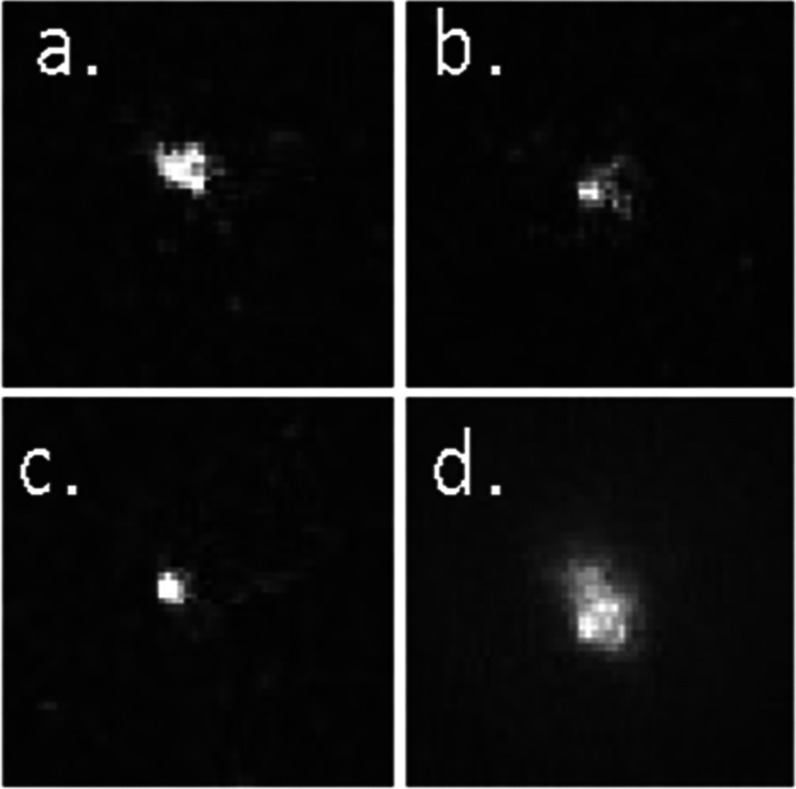
GNSs in 5% Gel-DES after 24 h of swelling. The luminescence is detected using an EMCCD camera and promoted by two-photon excitation at 800 nm at 10 mW on the sample. (a–c) Illustrative frames (out of 1000 frames acquired) at three arbitrary times; (d) average intensity over all frames. Field of view, 16 × 16 μm^2^.

In 1000 frames stack of images, GNSs appear to diffuse in the focal plane, as shown in Figure 9 in panels a–c for three exemplificatory time points. Panel d shows an intensity projection for all of the 1000 frames, showing that many particles are moving in and out of the excitation volume during the acquisition.

GNS diffusion is a consequence of the photothermal effect primed by the nanoparticles, and it becomes more and more pronounced at increasing laser power. The local temperature increase can promote a thermophoretic force that recruits GNS from the surrounding of the excitation spot region, thus increasing the local concentration, as shown in Figure 9. The observed effect might be exploited to drive a high concentration of nanoparticles in the selected regions of the hydrogel where, for instance, cells are present in order to induce local heating and/or release of drug loaded to the GNSs.

## Conclusions

DES resulted an effective cross-linking agent for the production of gelatin-based hydrogels; in particular, the 5% gelatin-DES hydrogels resulted in a suitable 3D scaffold for chondrocyte adhesion and spreading. Since cartilage tissue possesses a limited self-repair ability, the identification of DES-gelatin hydrogel as a suitable scaffold able to sustain chondrocyte culture may open a new perspective toward the development of a 3D platform for cartilage tissue engineering, still an ongoing challenge.

Moreover, diffusion, uptake, and release properties of the 5% DES-gelatin hydrogel suggest that it deserves further studies as a drug delivery system, exploiting its ability of release only upon *in vivo* degradation.

Interestingly, 5% Gel-DES hydrogel can also be considered as a smart material by incorporating gold nanoparticles of asymmetric shapes that absorb NIR light during the cross-linking process. A temperature-induced diffusion can then be used to obtain an increase in nanoparticle concentration that acts as hot spots in the gel. This application can pave the way to selective apoptosis of cells grown in the hydrogel by exploiting the photothermal effect.

## Materials and Methods

### Materials

All chemicals were purchased from Sigma-Aldrich and used without any further purification. Gelatin from porcine skin is provided by Sigma-Aldrich, catalog no. G2500, CAS Number 9000-70-8. Freeze-drying was performed by a Christ alpha 1–2 freeze dryer (Christ, Osterode am Harz, Germany). All samples were immersed in liquid nitrogen for about 1 h before the drying procedure. *C. histolyticum* collagenase (type I, ≥125 cdu/mg) was purchased from Sigma-Aldrich. Human cartilage cells C28/I2 were a kind gift from Prof. Francesco Dell’Accio (Queen Mary University of London, London, UK).

### Scaffold Preparation

#### 10% (w/v) Hydrogel Preparation (10% Gel-DES)

Gelatin (200 mg) was dissolved in 0.05 M Na_2_CO_3_/NaHCO_3_ buffer solution (pH 9.3, 2 mL) at 40 °C. After complete dissolution, DES (5 μL) was added. Gelatin was reacted for 90 min at 40 °C and then cooled to room temperature until complete gelation. Hydrogels were freeze-dried for 72 h.

#### 5% Hydrogel Preparation (5% Gel-DES)

Gelatin (100 mg) was dissolved in 0.05 M Na_2_CO_3_/NaHCO_3_ buffer solution (pH 9.3, 2 mL) at 40 °C. After complete dissolution, DES (5 μL) was added. Gelatin was reacted for 90 min at 40 °C and then cooled to room temperature until complete gelation. Hydrogels were freeze-dried for 72 h.

### Physico-Chemical Characterization

#### FTIR Analysis

FTIR spectra were collected in attenuated total reflection (ATR), as previously described.^31^ In particular, samples were forced into close contact with the diamond ATR crystal using the clamp arm assembly of the device (Quest, Specac), and the spectra were recorded using the Varian 670-IR spectrometer (Varian Australia Pty Ltd.). The following conditions were employed: 2 cm^–1^ spectral resolution, scan speed of 25 kHz, 512 scan conditions, triangular apodization, and a nitrogen-cooled mercury cadmium telluride detector. Gelatin absorption spectra were normalized at the same Amide I band area. FTIR measurements and spectral analyses were performed with the Resolutions-Pro software (Varian Australia Pty Ltd.).

#### Scanning Electron Microscopy Analysis

Morphological analysis was performed by means of scanning electron microscopy (SEM).

Freeze-dried hydrogel samples (1 g) were cut, placed with a conductive carbon tape onto standard SEM stubs, and sputter-coated with a 10 nm gold film, and the upper and inner surface were analyzed using a field-emission scanning electron microscope (FE-SEM) ZEISS GeminiSEM 500, operating at 2 kV accelerating voltage, using In-lens SE-detector configuration.

### Swelling Studies

Dynamic swelling measurements were made by gravimetric analysis. Three replicas of freeze-dried 5 and 10% Gel-DES specimens (ca. 1 g in weight) were soaked in 40 mL of deionized water at 25 °C. The swollen gels were periodically removed from water, blotted with the filter paper, weighed on an analytical balance (Analytical Balance 220 g × 0.1 mg, Radwag AS 220/C/2), and returned to the swelling medium till the equilibrium was reached.

SD_*t*_ (in g of water absorbed/g of dry hydrogel) was calculated using eq 1 and reported as a function of time
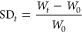
1where *W*_*t*_ is the weight of swelling hydrogel at different times and *W*_0_ is the dry weight of the gel. At long times, the ESD is reached. It is possible to fit the swelling kinetics by the integration of the Berens-Hopfenberg differential eq 2 that gives the characteristic swelling time of the hydrogel^26^
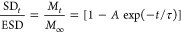
2where *M*_*t*_ is the amount of adsorbed water (*W*_*t*_ – W_0_), *M*_°_ is the adsorbed water at equilibrium (*W*_°_ – *W*_0_), and τ is the characteristic time of the kinetic.

When only the first part of the swelling curve is considered (SD_*t*_/ESD ≤ 0.6), it is possible to determine the mechanism of water diffusion inside the hydrogel from the value of the exponent of eq 3(27)
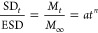
3where *n* = 0.5 accounts for Fickian (normal) water diffusion, when the diffusion rate of the penetrant is much lower than that in polymer relaxation, and *n* = 1 shows a transport (i.e., polymer relaxation controls the water diffusion into the network). Values of *n* between 0.5 and 1 indicate non-Fickian or anomalous transport, when both diffusion and polymer relaxation control water penetration into the network.

The EWC was calculated using eq 4(32)
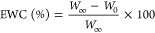
4where *W*_∞_ is the swelling weight of the sample at equilibrium and *W*_0_ is the dry weight of the gel.^33^

### Biological Characterization

#### Collagenase Assay

Hydrogel specimens (1 g in weight) were incubated with 1 mL of Tris–HCl buffer (0.1 M; pH 7.4) and CaCl_2_ (0.05 M) at 37 °C for 1 h, and 1 mL of *C. histolyticum* collagenase solution (0.5 mg/mL in Tris–HCl 0.1 M, at pH 7.4) was added to samples. All samples were kept at 37 °C, and the degradation was determined by gravimetric analysis.^34^

#### Cell Culture

Human cartilage cells C28/I2 were cultured in Dulbecco’s modified Eagle’s medium/Ham’s F-12 (DMEM/F-12, BioWest, USA) supplemented with 10% heat-inactivated fetal bovine serum (FBS, Euroclone S.p.A., Pero, Italy), 100 U/mL penicillin, 100 mg/mL streptomycin, 4 mM l-glutamine, and 17 mM d-glucose (BioWest, USA). C28/I2 cells were maintained at 37 °C in a humidified atmosphere with 5% CO_2_ in 100 mm Petri dishes. The medium was changed every 3 days. HEK293 cells, an embryonic renal immortalized cell line, were cultured in 100 mm Petri dishes using DMEM supplemented with 10% FBS, 100 U/mL penicillin, 100 mg/mL streptomycin, and 4 mM l-glutamine. HEK293 cells were maintained at 37 °C in a humidified atmosphere with 5% CO_2_. Medium was changed every 3 days. Cells were cultured in monolayers, grown sub-confluence (80%), and passaged at a ratio of 1:8.

#### Cell Inoculation

Cell inoculation into hydrogel cells was obtained after confluent monolayer cultures release by Trypsin–EDTA, and cells were counted and resuspended in growth medium at a density of 5 × 10^6^ cells/mL. Before cell seeding, freeze-dried hydrogels were sterilized in 75% aq. ethanol for 20 min, followed by 30 min UV and several washing with 1 mL PBS. After sterilization, hydrogels were equilibrated in 1 mL of culture medium (DMEM/F-12) for 12 h. All sponges were aspirated dry prior to cell seeding and disposed in a 24-multiwell plate. 200 μL of C28/I2 or HEK293 suspension (5 × 10^6^ cells/mL) per specimen were added to the wells, and cells were allowed to attach for 4 h at 37 °C with 5% CO_2_ in a humidified atmosphere. Subsequently, 1 mL of culture medium was added in each well. The culture was conducted for 2 weeks, maintained at 37 °C in a humidified atmosphere with 5% CO_2_, and medium was changed twice a week. Cells were observed daily using an inverted microscope (CK40, Olympus, Tokyo, Japan).

#### Immunofluorescence Analysis

After 2 weeks of culture, gelatin hydrogels containing cells were fixed with PBS-4% paraformaldehyde for 2 h at 4 °C and washed twice with PBS. Afterward, using a vibratome (VT1000S, Leica, Wetzlar, Germany), gelatin hydrogels were cut into 300 sections (300–100 μm thick), depending on the hydrogel composition. The gel sections were permeabilized with 1 mL of 0.1% Triton X-100 in PBS for 15 min, blocked with 1 mL of 1% BSA/PBS for 45 min, washed four times with 1% BSA/PBS, and stained with 1 μL of Phalloidin-TRITC (Sigma-Aldrich, Saint-Louis, Missouri, USA) in 1 mL of 1% BSA/PBS for 1 h. After five washing with PBS, DNA was stained using DAPI (Sigma-Aldrich, Saint-Louis, Missouri, USA), and sections were washed four times with PBS. Finally, gel sections were mounted using Eukitt quick-hardening mounting medium (Sigma-Aldrich, Saint-Louis, Missouri, USA).

Fluorescence imaging of the sections was performed using a scanning microscope exploiting two photon excitation by a home assembled setup.^35^ Briefly, a MaiTai Pro HP Titanium:Sapphire laser (Spectra Physics, Mountain View, Ca, USA) beam at 800 nm has been fed through a scanning head (FluoView 300, Olympus, Tokyo, Jp) and focalized on the sample by an IR optimized objective (25× XLplan, NA = 1.05, Olympus, Tokio, Jp). The fluorescence signal was collected in the non-descanned mode and separated by dichroic and band pass filters in front of the detectors (for DAPI, 485/30 nm, for TRITC, 600/40 nm Chroma Techn. Brattelboro, VT).

### Drug Release Studies

#### Rhodamine Loading Study

Fresh made hydrogels (5% Gel-DES, obtained from 100 mg of gelatin) were placed in 3 mL of Rhodamine-6G solution (7 μM) in milliQ water. Every 20 min, the absorbance of the solution was measured by means of a spectrophotometer, and the solution concentration was obtained according to the Lambert–Beer law *A* = ε *c l*, where ε = 116 000 M^–1^ cm^–1^ is the Rhodamine extinction molar coefficient, *l* = 0.2 cm is the cuvette path length, and *c* is the molar solution concentration.

#### Rhodamine Release Study

The release kinetics is followed by soaking the hydrogel samples, containing Rhodamine solution, in 3 mL of milliQ water. The absorbance was measured at 15 min intervals, changing the sample water every time under the same condition previously cited (molar extinction and path length). Data were reported normalized to the equilibrium value.

### Diffusion Studies

Diffusion of small fluorescent probes (Rhodamine 6G and GFP, Sigma Chemical Co.) was characterized by the FCS technique. Fluorescent nanobeads of average diameter of 20 nm were purchased by Life Science. GNSs were synthesized by a lauryl-sulfobetaine (LSB)-driven seed growth, as reported elsewhere (0.35 M LSB concentration). The GNSs have average branch sizes of (53.5 ± 1.2) nm and (9.5 ± 0.2) nm, yielding a plasmonic absorption band centered at 780 nm.^29^

The home-built FCS setup has been described elsewhere.^28^ Briefly, the output of a Titanium:Sapphire laser beam (Tsunami, Spectra Physics, Mountain View, Ca, USA) at 800 nm for Rhodamine or 890 nm for GFP excitation is focused by a water immersion objective (Plan Apochromat 60x water objective NA = 1.2, Nikon, Japan) mounted on an inverted TE300 Nikon (Japan) microscope. The fluorescence is collected in epifluorescence geometry, separated from excitation by a set of dichroic mirrors and band pass filters (560/40 for Rhodamine and 535/50 for GFP), split in two by a 50% cube, and focused on the active area of two APD detector set at 90° in cross-correction geometry. This avoids artifacts due to detectors dead time or after pulsing. The digital signal from the APDs is fed into a two-channel autocorrelation board (ALV 7002/USB, ALV-Laser Vertriebsgesellschaft mbh, Langen, D) inserted in a personal computer and stored for analysis.

The laser beam is tightly focused in a small volume (≃μm^3^), and if the concentration of the fluorescent probe is sufficiently low (1–100 nM range), fluorescence fluctuations arising from molecules moving in and out across the excitation volume can be easily detected. The temporal behavior of the signal fluctuations can give information on the diffusion characteristics of the probe through the fluorescence ACF, defined by eq 5
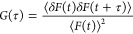
5where δ*F*(*t*) = *F*(*t*) – ⟨*F*(*t*)⟩ is the fluorescence fluctuation with respect to the average value and τ is the time lag. If only diffusing motions are taken into account as in the case treated here, the explicit expression for the ACF is given by eq 6(36)
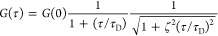
6where τ_D_ = ϖ_0_^2^/8*D* is the characteristic diffusion time through the beam waist (ϖ_0_) for a species with diffusion coefficient *D*, ζ^2^ is the axial ratio, and *G*(0) the zero time lag value of the autocorrelation. Fitting of the ACF is accomplished using OriginLab 9 Software.

For measuring longer diffusing species such as gold nanoparticles in the hydrogel, an EMCCD camera (Cascade II, Photometrics, Tucson, AZ) has been connected to the previous setup through a dedicated port, diverting the signal from the APD detectors to the EMCCD. The EMCCD has a resolution of 512 × 512 pixels, a pixel size of 16 μm, and a shortest acquisition time of 4 ms. Stacks of images have been acquired with a typical frame rate of 30 fps and a typical field of view of 16 × 16 μm^2^.

When needed, a confocal scanning Leica SP5 microscope has been used employing the 488 nm excitation wavelength of an argon laser.

## Supporting Information Available

Magnified SEM images, C28/IC2, and HEK293 micrograph in the 10% Gel-DES (PDF)
Cell behavior when embedded in the 5% Gel-DES with C28/I2 cells (magnification 28 μm); 5% Gel-DES with C28/I2 cells (magnification 57 μm); 5% Gel-DES with HEK293 cells (magnification 23 μm); and 5% Gel-DES with HEK293 cells (magnification 57 μm) (ZIP)

## Abbreviations

ACF: autocorrelation function
AP: 1-aminopropane
DES: 3,4-diethoxy-3-cyclobutene-1,2-dione or diethyl squarate
APD: avalanche photodiode
ATR: attenuated total reflection
EMCCD: electron multiplying charge-coupled device
FCF: fluorescence correlation spectroscopy
FE-SEM: field-emission scanning electron microscopy
FTIR: Fourier-transform infrared spectroscopy
GFP: green fluorescent protein
GNS: gold nanostars
LSB: lauryl-sulfobetaine
NIR: near-infrared
