# Supplementary material for: Squarate Cross-Linked Gelatin Hydrogels as Three-Dimensional Scaffolds for Biomedical Applications
Source: Langmuir. 2021 Nov 22;37(48):14050–8. doi: 10.1021/acs.langmuir.1c02080 (PMC8655982; doi:10.1021/acs.langmuir.1c02080)

SUPPORTING INFORMATION

Squarate cross-linked gelatin hydrogels as 3D scaffolds for biomedical applications

Simone Stucchi,[§,†,‡] *Danilo Colombo,* [§‡,¶] *Roberto Guizzardi,* [§,∫] *Alessia D'Aloia,*[§] *Maddalena Collini,*[⊥, ⌘] *Margaux Bouzin,*[⊥] *Barbara Costa,*[§] *Michela Ceriani,*[§] *Antonino Natalello,*[§] *Piersandro Pallavicini,*[#] *Laura Cipolla*[§] *

[§]Dept. of Biotechnology and Biosciences, University of Milano - Bicocca, P.zza della Scienza 2, 20126 Milano-Italy

[⊥]Dept. of Physics "Giuseppe Occhialini", University of Milano-Bicocca, P.zza della Scienza 3, 20126 Milano-Italy

[⌘]Nanomedicine Center, University of Milano-Bicocca, P.zza della Scienza 3, 20126 Milano-Italy

[#]Dept. of Chemistry, Università degli Studi di Pavia, Viale Taramelli 12, 27100, Pavia-Italy

* Corresponding Author: Prof. Laura Cipolla, laura.cipolla@unimib.it

Table of contents

Figure S1. a) Longitudinal portion SEM images of Gel-DES 5%. b) Longitudinal portion SEM images of Gel-DES 10%

Figure S2. C28/I2 chondrocytes (top) and HEK293 (bottom) cells plated on 10% Gel-DES. C28/I2 and HEK293 cells were plated on 10% Gel-DES at high density and were growth for 2 weeks. Then hydrogels were cutted into spieciments and stained with Phalloidin-TRITC (Green) and DAPI (Blue). Images were taken by a scanning microscope exploiting two photon excitation. A) Representative images of C28/I2 cells into 10% Gel-DES. B) Representative images of HEK293 cells into 10% Gel-DES.

The following files are also available free of charge:

Supporting movie S1 (.mov) 5% Gel-DES with C28/I2 cells (magnification 28 micron)

Supporting movie S2 (.mov) 5% Gel-DES with C28/I2 cells (magnification 57 micron)

Supporting movie S3 (.mov) 5% Gel-DES with HEK293 cells (magnification 23 micron)

Supporting movie S4 (.mov) 5% Gel-DES with HEK293 cells (magnification 57 micron)

Figure S1. a) Longitudinal portion SEM images of Gel-DES 5%. b) Longitudinal portion SEM images of Gel-DES 10%

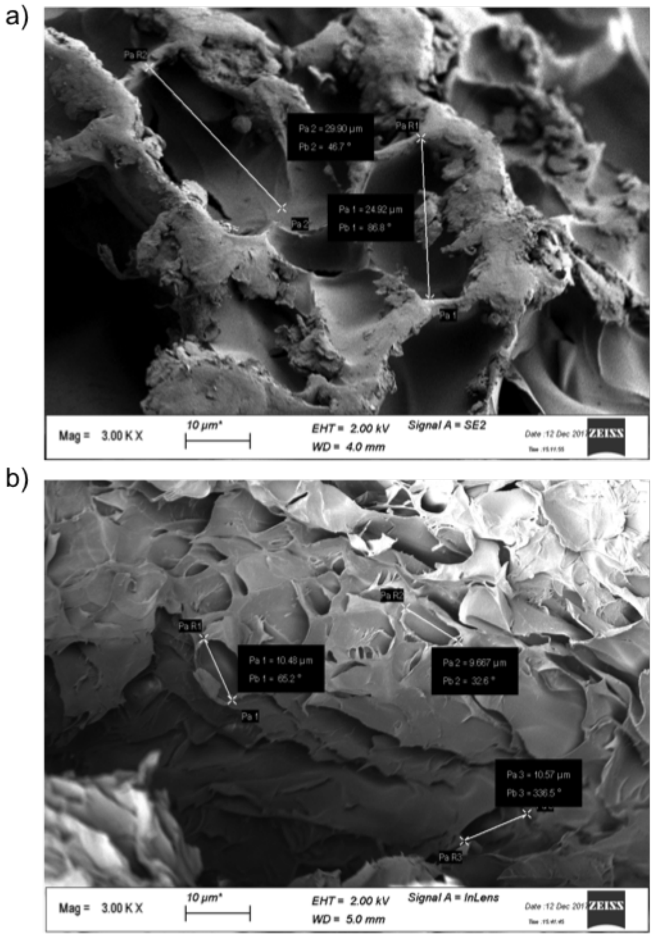

Figure S2. C28/I2 chondrocytes (top) and HEK293 (bottom) cells plated on 10% Gel-DES. C28/I2 and HEK293 cells were plated on 10% Gel-DES at high density and were growth for 2 weeks. Then hydrogels were cut into specimens and stained with Phalloidin-TRITC (Green) and DAPI (Blue). Images were taken by a scanning microscope exploiting two photon excitation. Bar size is 71 μm for left panels, 47 μm and 35 μm for the right panels, up and down, respectively.

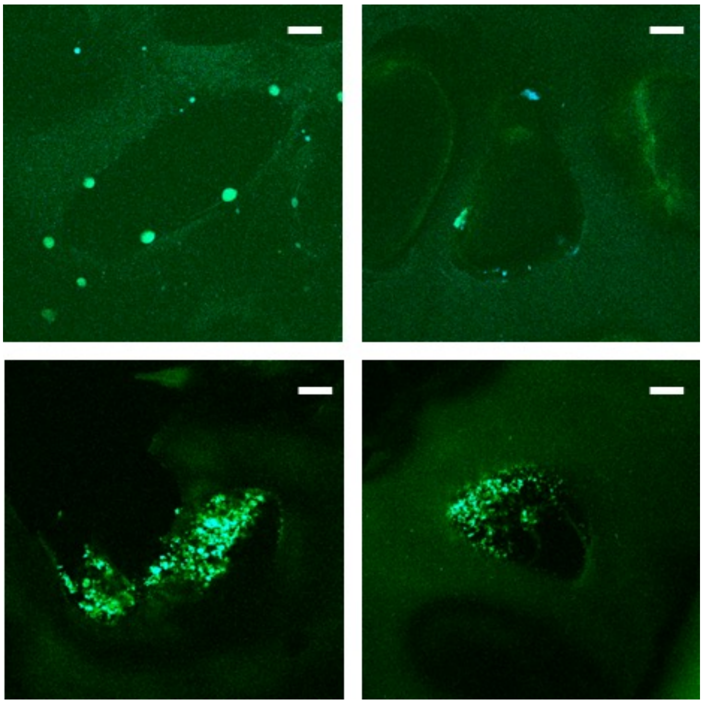

Supplement: Supplementary file 1 — la1c02080_si_001.pdf [file la1c02080_si_001.pdf]
